# The PrecisionTox chemical library: creation of a chemical collection to discover evolutionary conserved biomolecular signatures of toxicity

**DOI:** 10.1093/toxsci/kfaf126

**Published:** 2025-11-04

**Authors:** Rubén Martínez, Juan Carlos González-Sánchez, Stavroula I Sampani, Stefan Scholz, Beate I Escher, Luise Henneberger, Julia Huchthausen, Maurice Whelan, Thomas Dickmeis, Carsten Weiss, John K Colbourne, Jonathan H Freedman

**Affiliations:** Leitat Technological Center, Ecotoxicology & Biodegradability Unit, Terrassa, 08225, Spain; Biochemistry Center (BZH), Heidelberg University, Heidelberg, 69120, Germany; European Commission, Joint Research Centre (JRC), Ispra (VA), 21027, Italy; Department of Ecotoxicology, Helmholtz Centre for Environmental Research—UFZ, Leipzig, 04318, Germany; Department of Cell Toxicology, Helmholtz Centre for Environmental Research—UFZ, Leipzig, 04318, Germany; Department of Cell Toxicology, Helmholtz Centre for Environmental Research—UFZ, Leipzig, 04318, Germany; Department of Cell Toxicology, Helmholtz Centre for Environmental Research—UFZ, Leipzig, 04318, Germany; European Commission, Joint Research Centre (JRC), Ispra (VA), 21027, Italy; Institute of Biological and Chemical Systems—Biological Information Processing, Karlsruhe Institute of Technology, Eggenstein-Leopoldshafen, 76344, Germany; Institute of Biological and Chemical Systems—Biological Information Processing, Karlsruhe Institute of Technology, Eggenstein-Leopoldshafen, 76344, Germany; Centre for Environmental Research and Justice and School of Biosciences, University of Birmingham, Birmingham, B15 2TT, UK; Centre for Environmental Research and Justice and School of Biosciences, University of Birmingham, Birmingham, B15 2TT, UK; Gillings School of Global Public Health, The University of North Carolina at Chapel Hill, Chapel Hill, NC 27599, United States

**Keywords:** chemical libraries, new approach methodologies, adverse outcome pathways, physicochemical properties, baseline-toxicity prediction model

## Abstract

The large number and diversity of chemicals currently in use present significant challenges in assessing their human and environmental health risks due to a paucity of toxicological data. To address this shortage, high-throughput screening technologies are used to rapidly evaluate the toxicity of these chemicals. Suitable chemical libraries are crucial to evaluate the performance of these technologies and generate the cognate toxicity data. Unlike traditional chemical libraries designed for specific disease targets or receptor interactions, the PrecisionTox collection prioritizes diversity in targets and mechanisms of toxicity to ensure broad applicability in toxicity predictions to test the concept of phylotoxicology. Phylotoxicology proposes that mechanisms of toxicity are evolutionarily conserved among distantly related species. Furthermore, the application of phylotoxicology can contribute to the reduction of mammalian species in toxicity testing. Here, an approach for generating a chemical library based on chemical properties—physicochemical, biomolecular, and toxicological—as well as practical considerations, including compound availability, cost, purity, and shipping regulations, is reported. From an initial pool of over 1,500 nominees, a set of 200 chemicals was selected based on multiple criteria, including organ toxicity, environmental exposure, structure, modes of action, and toxicological relevance. Additionally, information on baseline toxicity, Absorption, Distribution, Metabolism, and Excretion properties and utility for in vitro testing was collected. This work underscores the necessity of thoughtful chemical selection to refine toxicological models, improve hazard identification, and support regulatory efforts to protect human and environmental health.

An estimated 350,000 chemicals are being used both commercially and globally (Wang et al. 2020). Humans and other species are exposed to many of these chemicals, for which frequently little to no toxicological information is available (Wang et al. 2020; Persson et al. 2022). To address this gap in chemical risk and hazard information, methods have been developed to measure a variety of toxicological endpoints using in vivo and in vitro high-throughput screening (HTS) technologies (Richard et al. 2016). At the same time, New Approach Methodologies (NAMs) offer a sustainable alternative to animal testing (Colbourne et al. 2025). NAMs enhance human relevance and can reduce costs and resources, facilitating the rapid screening of large numbers of chemicals and the identification of potential hazards. In vitro HTS platforms acquire data at rates of millions of chemicals per week (Wunder et al. 2008), whereas in vivo platforms can examine tens to a few thousand chemicals per week (Murphey and Zon 2006).

The ability to rapidly measure toxicological effects of large numbers of chemicals using different endpoints and analyze the cognate data continues to develop. Chemical libraries have been created using an array of criteria to test toxicological endpoints. For instance, compounds have been selected to target specific organs (liver, kidney, heart), disease endpoints (cancer, developmental neurotoxicity, fibrosis), receptors or enzymes (serotonin transporters, G-protein coupled receptors, acetylcholinesterase), biological pathways (fatty acid oxidation, protein kinase, endocrine/androgen), chemical classes (metal/metalloids, pesticides), or chemical structures (bisphenols, perfluorooctanoic acids, polycyclic aromatic hydrocarbons) (Richard et al. 2021).

A variety of approaches have been used to collect compounds for testing with known or anticipated toxicities into training sets or reference libraries. The simplest approach is to use chemicals contained in predefined lists. Agencies throughout the world, such as the Joint Research Centre (JRC), the Organization for Economic Co-operation and Development (OECD), and ToxCast and the Partnership for the Assessment of Risks from Chemicals (PARC) (Snapkow et al. 2024), are constantly assembling lists of chemicals that meet different criteria (e.g. JRC validation sets, OECD Test Guidelines or Guidance documents). Other methods include literature mining to identify compounds with specific toxicological characteristics (Canham et al. 2020), generating and subsequently synthesizing compounds based on Quantitative structure–activity relationship (QSAR) models, and synthesizing congeners of known drugs (Ashenden 2018). The number of compounds in these libraries can range from 10’s for small projects to >10,000 for larger projects such as Tox21 (Richard et al. 2021).

PrecisionTox is an international consortium formed under the European Commission Horizon 2020 program with the goal of identifying toxicity pathways and cognate biomarkers associated with chemical exposure using NAMs (PrecisionTox Consortium 2023). To achieve this goal, PrecisionTox is testing the concept of phylotoxicology, which proposes that many biomolecular and toxicological responses to chemical exposure are shared among distantly related species by evolutionarily conserved processes and pathways. Thus, screening chemicals in a suite of phylogenetically diverse species, including biomedical and ecologically relevant models, can be used to assess chemical hazards to wildlife and humans without the use of traditional mammalian species (Colbourne et al. 2022). To evaluate this concept, a collection of 200 compounds was assembled and tested in a human cell line, *Caenorhabditis elegans*, *Daphnia magna*, *Drosophila melanogaster*, and embryos of *Danio rerio* and *Xenopus laevis*. Unlike other libraries and smaller test sets, the defining characteristic of the PrecisionTox collection is its diversity in target organs, receptor binding, affected biological pathways, and chemical class. Consequently, there are hundreds of thousands of potential candidates.

To choose 200 chemicals for this collection, a chemical selection strategy was established to select compounds that target specific organs, have environmental relevance, particular chemical structures, and/or modes of action (MoA). This collection was designed to be a community resource that contains potential “MOA anchor chemicals” (OECD 2017). Here, the rationale for choosing chemicals for the PrecisionTox collection is presented. To define the characteristics of this collection, experimental and theoretical data on physicochemical, toxicological, and Absorption, Distribution, Metabolism, and Excretion (ADME) characteristics were collected. For easier access to this information, the data has been incorporated into the PrecisionTox Data Visualization Tool. Ultimately, this chemical collection and cognate data will be combined with transcriptomic- and metabolomic-based toxicological data from the 6 alternative test models to test the phylotoxicology hypothesis with the goal of advancing the use of NAMs (PrecisionTox Consortium 2023).

## Materials and methods

### Selection of chemicals for the PrecisionTox collection

Several overall considerations were maintained during the chemical selection process. This included identifying reference compounds, well-studied chemicals with large amounts of associated toxicological and mechanistic data. Similarly, chemicals with limited or poorly understood mechanistic data were selected to test future toxicity predictions. General and taxon-specific toxicants, which targeted taxa from various parts of the phylogenetic tree, were selected. These were used to test the phylotoxicology hypothesis. Assembling the chemical collection also considered identifying and addressing issues in the logistics of obtaining and shipping chemicals to international partners. Additionally, it enabled the various groups in PrecisionTox to develop Standard Operating Procedures for toxicity testing, database development, and analysis processes.

#### Chemical nominations

The first step in building the chemical collection was to define its chemical space. From discussions within PrecisionTox, stakeholders, and partner EU projects, including Animal-free Safety Assessment of Chemicals: Project Cluster for Implementation of Novel Strategies (ASPIS), priority was given to several chemical groups (Table 1). ASPIS has a strong focus on hepatotoxicants, nephrotoxicants, and developmental neurotoxicants (DNTs). Thus, the phylogenetic approach of PrecisionTox is synergistic and mutually beneficial to the project cluster by including these chemical groups. Chemical structure-specific toxicants were selected, including conazoles, acrylamides, and imidazoles. This group of chemicals was selected as part of collaborative case-study projects investigating the grouping of substances based on bioactivity data from multiple testing platforms between PrecisionTox, chemical regulatory agencies (acrylamides and imidazoles), and PARC (conazoles). Furthermore, environmentally relevant chemicals were prioritized (Finckh et al. 2022; Scholz et al. 2022; Finckh et al. 2024). To maintain diversity in the PrecisionTox collection, a target number of 20 to 30 chemicals per group was chosen.

**Table 1.** Groups used in chemical selections.

Organ-specific toxicants	Reference
Hepatotoxicants	(Albrecht et al. 2019)
Nephrotoxicants	(Su et al. 2016)
Developmental neurotoxicants	(Aschner et al. 2017; Blum et al. 2023)
Cardiotoxicants	(Sirenko et al. 2017)
**Chemical class-specific toxicants**	
Conazoles	(Peyton et al. 2015)
Acrylamides	(Koszucka et al. 2020)
Imidazoles	(Sharma et al. 2021)
**Environmentally relevant chemicals**	(Finckh et al. 2022; Scholz et al. 2022; Finckh et al. 2024)

**Fig. 1.**
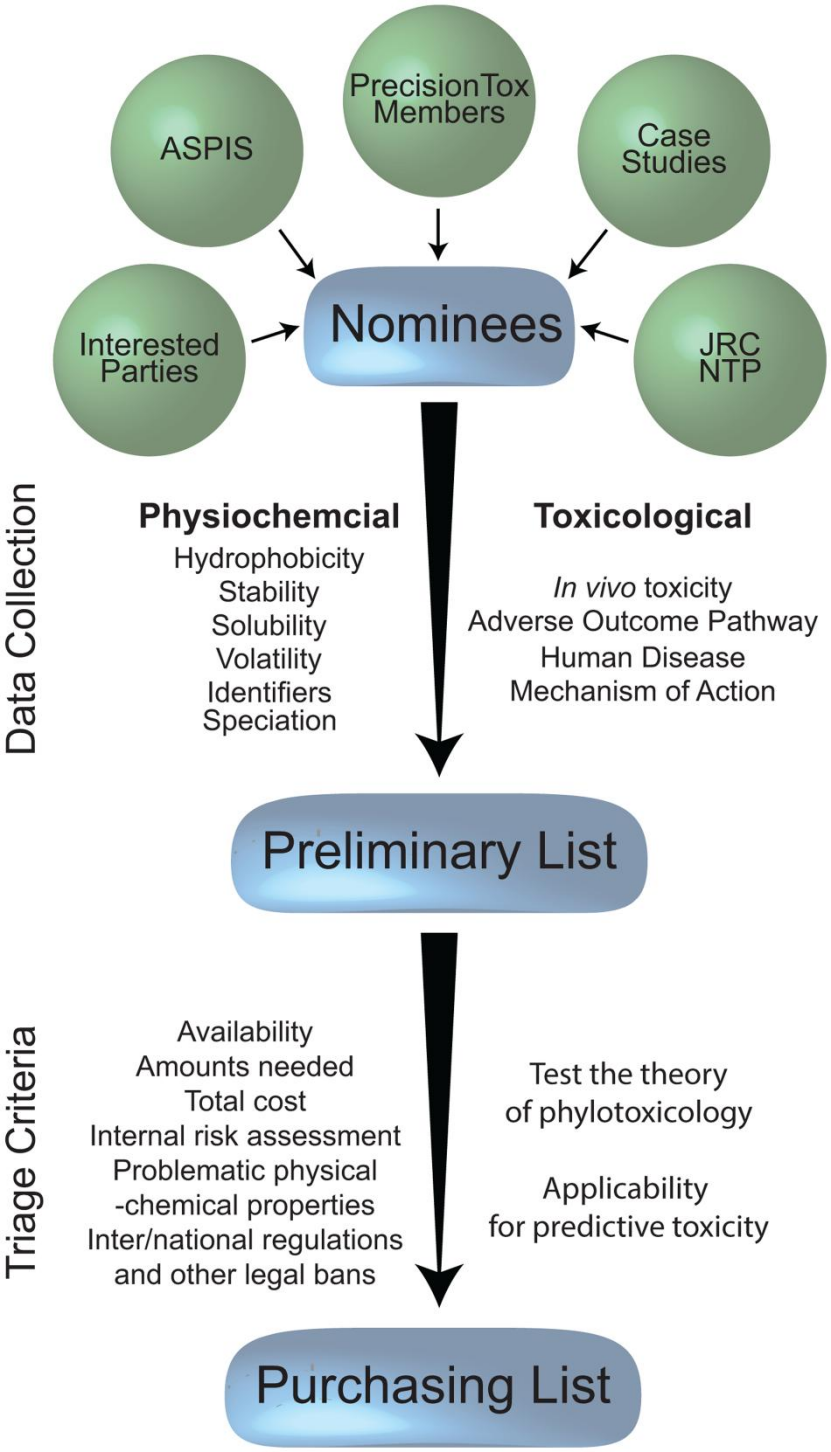
Chemical selection process. Diagram of the PrecisionTox chemical collection selection process. Briefly, chemical nominees are obtained from multiple sources from within and without the consortium. After collecting physicochemical and toxicological data, nominees are selected for purchase based on the triage criteria.

After defining the toxicological space in Table 1, the next step was to compile chemical nominees for inclusion into the collection. Nominees were obtained from consortium members and by manually mining available datasets from the US National Toxicology Program, the JRC (Sund and Deceuninck 2021), and the literature. Additional nominees were obtained from other international projects involved in developing NAMs and non-animal testing paradigms (Westmoreland et al. 2022; De Castelbajac et al. 2023). These were included to maximize collaborative efforts among these projects (Fig. 1). Some chemicals were excluded from nomination because of the biological limitations of the PrecisionTox test organisms. For example, pulmonary and dermal toxicants were not considered because none of the test species have systems analogous to lungs or mammalian skin.

#### Triaging process

Preliminary lists of several hundred chemical nominees within each group of toxicants were subjected to a tiered selection approach. First, following the compilation of physicochemical properties of the nominees, those with physicochemical properties outside the applicability domain or resulting in reduced bioavailability in the bioassays were removed. Those properties included volatility and hydrophobicity, as well as stability and solubility in aqueous media and/or DMSO. Volatile chemicals were removed based on an empirical threshold of ionization-corrected air–water partition constant *D*_aw_ >10^−4^ (Escher et al. 2019a). Hydrophobic chemicals with log *D*_lip/w_ (pH 7.4) > 4 were avoided to meet the criteria of in vitro assays, as they are challenging to dose in the small organism bioassays (see below). These criteria avoided potential declines in chemical test concentrations during exposure. Exceptions were made, however, for the inclusion of some compounds with a specific MoA if representatives within the defined range of physicochemical properties were unavailable. The first triaging step excluded approximately 75% of the original nominees.

The second step of the triaging process focused on pragmatic logistic aspects of obtaining and shipping of the compounds: Availability, amounts, cost, purity, inter/national regulations, and human safety. After compiling logistic data including potential manufacturers/providers, price per gram, purity, UN numbers, transport toxicity and packaging groups, International Air Transport Association classification, and exempted amounts allowed for shipping, the following restrictions were implemented. Generally, only chemicals with purities >98% were considered. Based on the available experimental and theoretical toxicity information and the experimental design for each model species, calculations were performed to determine the amount of each chemical required. Depending on amounts needed and cost, economically non-affordable chemicals were omitted, and a limit of 1,000€ per chemical was imposed for practical reasons. Additionally, some chemicals were not commercially available nor in the required amounts or formats. Nominees were also discarded if they were classified as a prohibited/restricted chemical (e.g. potential for misuse or addiction, chemical warfare agents, explosives). Chemicals considered too toxic to be used in the laboratory without special training and precautions (e.g. gases, potent inhalable carcinogens, potent toxicants, etc.) were also discarded. A summary of the exclusion list criteria is presented in Fig. 1. The second level of triaging excluded an additional ∼45% of the candidates from the first step.

#### Final selection and considerations

For several of the chemical groups, the first 2 triaging processes reduced the nominees to 20 to 30 compounds. In these cases, no further selection was necessary. In situations where a larger number of nominees remained, a third triaging step was introduced. Chemicals were selected based on additional toxicological parameters and their applicability in the identification of conserved toxicity pathways and biomarkers. These parameters included relevance to human exposure, in vivo and in vitro toxicities in multiple species, chemical transformation via metabolism, and molecular mechanism of toxicity. Information on these endpoints was collected by literature mining and from multiple databases (see Supplementary Material 2). Chemical identifiers including chemical name, CAS number, DSSTox substance identifier (DTXSID), or InChiKey were used in database searches and literature mining.

To maintain diversity in chemical space, only 1 or 2 chemicals that affect a specific mechanism of toxicity, Key Event, receptor, or Molecular Initiating Event per group were included. Finally, higher priorities for inclusion were given to chemicals identified in the human exposome (Barupal and Fiehn 2019; Neveu et al. 2020).

It was deemed crucial to minimize possible bias during the preparation of sample material for toxicity testing and omic analyses (PrecisionTox Consortium 2023). Therefore, each chemical used in the project was purchased from a single vendor and the same manufacturer’s lot. To maintain consistency across the laboratories, chemicals were shipped from the manufacturer to a single location, where they were stored under controlled conditions. Based on the solubility and stability of the test compound, chemical aliquots were distributed as dry powder or as a DMSO stock solution (see Supplementary Material 1) to the toxicity testing groups in Europe, United Kingdom, and the United States to allow them to expose each corresponding model organism to the same chemical batch, including its possible impurities. As noted above, local and international shipping regulations/restrictions were also considered part of the triaging process and during the aliquoting process (e.g. maximum amount per vial allowed by regulation) (Fig. 1).

We included 3 known baseline toxicity compounds—N-methylaniline, diphenylamine, and butoxyethanol—in the compound selection (Kluver et al. 2016). They served as negative controls for specific, reactive, or uncoupling interactions. Furthermore, compounds selected for specific organ toxicity can serve as negative controls for other organ-specific interactions.

### Physicochemical properties

Physicochemical properties of each nominee were collected from public databases or calculated de novo using the methods and models described in Supplementary Material 3 using the cognate data listed in Supplementary Material 4. Physicochemical data were derived from various experimental and prediction sources potentially associated with uncertainty. Hence, for the interpretation of toxicity data derived from the nominees, the uncertainty and potentially a re-measurement of physicochemical data to avoid any bias in data interpretation have to be considered.

Henry coefficients (*K*_H_) were estimated using OPERA version 2.6 or obtained from the CompTox Chemicals Dashboard (Williams et al. 2021; US Environmental Protection Agency 2023). They were then converted to air–water partition coefficients (*K*_aw_) and corrected for ionization at a given pH to obtain the ionization-corrected air-water distribution ratio *D*_aw_ (pH).

Octanol–water partition constants (*K*_ow_) were retrieved from experimental data in original publications or CompTox. If experimental data were not available, the *K*_ow_ was predicted using a poly-parameter linear solvation energy relationship (LSER) using the UFZ-LSER database (Ulrich et al. 2021). Alternatively, the mean *K*_ow_ was determined from the predicted values using KOWWIN v1.67 and ACD/Labs consensus data obtained from CompTox.

Experimental acidity constants (p*K*_a_) were obtained from the literature or experimentally measured using a Sirius T3 titrator (Pion, Inc.) (Niu et al. 2022; Huchthausen et al. 2024). If experimental data were not available or the prediction indicated that speciation did not change in the pH range relevant for the bioassays, p*K*_a_ values were predicted with ACD pKa/GALAS (ACD/Percepta 2015). Fractions of all neutral or zwitterionic (= *f*_neutral_), negative, double negative, positive, or double positive species were calculated using the Henderson–Hasselbalch equation (Escher et al. 2020).

### Prediction of in vitro and in vivo properties

#### Membrane–water partition constants

The uptake of chemicals into biological membranes is important in understanding bioavailability and baseline toxicity, the minimal toxicity that every chemical exhibits (Huchthausen et al. 2024; Qin et al. 2024). The distribution ratio between liposomes, which are typically used as proxies of biological membranes, and water (*D*_lip/w_) is composed of the *K*_lip/w_ of all species, including neutral and multiple charged species that can be measured or predicted with LSER or from the *K*_ow_ (Lee et al. 2021; Ulrich et al. 2021).

#### Protein–water partition constants

Bovine serum albumin (BSA) serves as surrogate for protein binding in medium. If experimental data were available, the distribution ratio between BSA and water, *D*_BSA/w_ at pH 7 to 7.4, served as a proxy for protein binding. If experimental data were not available, the *K*_BSA/w_ of the neutral species was calculated with a LSER or was predicted from log *K*_ow_ (Endo and Goss 2011). The *K*_BSA/w_ of cations was assumed to be the same as for neutral chemicals. Because anionic chemicals have a higher affinity to BSA, the prediction model for the non-specific portion of the sorption isotherm of anionic polyfluoroalkyl substances was used (Qin et al. 2024). The *K*_BSA/w_ was fixed at 1.31 for hydrophilic chemicals (log *K*_ow_ < 2) (deBruyn and Gobas 2007).

For chemicals where experimental data or LSER parameters were unavailable for cellular proteins, the *K*_SP/w_ was predicted from *K*_ow_ (Endo et al. 2012). Anion binding to structural proteins (SPs) is weaker than to BSA, and equations analogous to those for *D*_BSA/w_ (pH) were used to predict *D*_SP/w_ (pH) (Henneberger et al. 2016).

#### In vitro distribution modeling to estimate in vitro chemical availability

Various in vitro chemical distribution models for cell-based bioassays exist (Proença et al. 2021). They typically describe processes of binding to medium components, plastic, and cells, as well as volatility. Volatile chemicals were not included in the PrecisionTox chemical collection. Media of the cellular bioassays are typically supplemented with 2% to 10% fetal bovine serum (FBS). Under such circumstances, binding to the plastic is negligible (Fischer et al. 2018). This is different for water-based media where dosing solutions may have to be renewed (depending on physicochemical properties) or concentrations measured (Fischer et al. 2018). The remaining processes are binding to medium components (proteins and lipids) and cells. The freely dissolved fraction *f*_free_ can be calculated with Equation 1


(1) $$f_{\text{free}}=\frac{1}{1{+D}_{\text{medium}/w}\times \frac{V_{\text{protein}+\text{lipid},\;\text{medium}}}{V_{\text{medium}}}+D_{\text{cell}/w}\times \frac{V_{\text{cell}}}{V_{\text{medium}}}}$$

The partition constant between medium components and water *D*_medium/w_ can be predicted by mass balance models assuming that proteins and lipids are the dominant sorptive phases. Their volume fractions of proteins (*VF*_protein, medium_) and lipids (*VF*_lipid, medium_) in the medium are considered in the mass balance equation (Equation 2). BSA is typically used as a surrogate for medium proteins.


(2) $$D_{\text{medium}/w}=D_{\text{BSA}/w}\times {\;\text{VF}}_{\text{protein},\;\text{medium}}+D_{\text{lip}/w}\times {\;\text{VF}}_{\text{lipid},\;\text{medium}}$$

The *VF*_protein, medium_ is the ratio of volume of proteins *V*_protein, medium_ to the sum of the volume of both sorptive phases, proteins, and lipids, *V*_protein+lipid, medium_ (Equation 3), and analogously for *VF*_lipid, medium_ (Equation 4)


(3) $${\;\text{VF}}_{\text{protein},\;\text{medium}}=\frac{V_{\text{protein},\;\text{medium}}}{V_{\text{protein}+\text{lipid},\;\text{medium}}}$$

(4) $${\;\text{VF}}_{\text{lipid},\;\text{medium}}=\frac{V_{\text{lipid},\;\text{medium}}}{V_{\text{protein}+\text{lipid},\;\text{medium}}}.$$

Analogously, *D*_cell/w_ can be predicted by Equation 5. The most abundant proteins in cells are SPs, for which muscle proteins are better surrogates than BSA (Henneberger et al. 2016). In cells, the volume of water *V*_water, cell_ is also considered.


(5) $$D_{\text{cell}/w}=D_{\text{SP}/w}\times \frac{V_{\text{protein},\;\text{cell}}}{V_{\text{cell}}}+D_{\text{lip}/w}\times \frac{V_{\text{lipid},\;\text{cell}}}{V_{\text{cell}}}+\frac{V_{\text{water},\;\text{cell}}}{V_{\text{cell}}}$$

### Baseline toxicity

The nominal baseline cytotoxicity concentration for mammalian cell lines can be calculated from the mass balance model from the critical baseline-toxic membrane concentration IC_10, membrane_ (Equation 6).


(6) $${\text{IC}}_{10,\;\text{nom},\;\text{baseline}}=\frac{{\text{IC}}_{10,\;\text{membrane}}}{D_{\text{lip}/w}}\times (1+D_{\text{BSA}/w}\times {\text{VF}}_{\text{protein},\;\text{medium}}+D_{\text{lip}/w}\times {\text{VF}}_{\text{lipid},\;\text{medium}}+D_{\text{cell}/w}\times \frac{V_{\text{cell}}}{V_{\text{bioassay}}})$$

IC_10, membrane_ varies little across species and cell types, but is dependent on the measured toxicity endpoint. The IC_10, membrane_ for cell lines used in PrecisionTox, for a 10% reduction of cell proliferation as endpoint, was estimated to be approximately 26 mmol/L_lip_ (Huchthausen et al. 2024). For a medium supplemented with 10% FBS, the volume fraction of proteins in medium, VF_protein, medium_, was 0.3% and of lipids. VF_lipid, medium_ was 0.007%. The volume fraction of proteins in cells VF_protein, cells_ was 3% and of lipids VF_lipid, cell_ was 0.5%, which are means of typical experimental data (Huchthausen et al. 2024).

The freely dissolved chemical concentration IC_10, free_ (Equation 7) can be used to compare the in vitro cytotoxicity with lethal effects in vivo.


(7) $${\text{IC}}_{10,\;\text{free}}=\frac{{\text{IC}}_{10,\;\text{membrane}}}{D_{\text{lip}/w}}$$

The baseline toxicity lethal concentration for 50% of the test organisms in vivo, LC_50_, was predicted using linear regression QSAR models given in Equation 8.


(8) $$-\mathrm{logL}C_{50}=\mathrm{a log}\;D_{\mathrm{lip}/w}+b$$

The use of the *D*_lip/w_ as a QSAR descriptor, rather than *K*_ow_, allows one to also apply QSAR to ionizable compounds.

The baseline toxicity prediction model for *C. elegans* was rescaled from a *K*_ow_-based model using of Equation 4 in Supplementary Material 3 and the descriptors are *a* = 0.81 and *b* = 1.15. The model for *D. magna* (*a* = 0.82, *b* = 1.48) was derived from the *K*_ow_-based model using 48-h LC_50_ (Zhao et al. 1998). The QSAR for *D. rerio* was directly developed with *D*_lip/w_ and has *a* = 0.99 and *b* = 0.78 (Kluver et al. 2019). There are no baseline toxicity prediction models for *X. laevis* and *Drosophila*. Therefore, experimental LC_50_ data for 22 chemicals in the PrecisionTox collection that were identified as baseline toxicants in both cell lines and zebrafish were used to derive new regressions. For both species, the baseline toxicants correlated linearly with the log *D*_lip/w_, yielding *a* = 0.61 and *b* = 2.12 (*r*^2^ = 0.690) for *X. laevis* (*n* = 22). For *Drosophila*, only the BMD_50_ (mol/L_feed_) of 11 baseline toxicants were causing effects resulting in a regression with *a* = 0.83 and *b* = 0.52 (*r*^2^ = 0.724). These QSARs are considered preliminary, but they serve to define the dosing concentrations in the PrecisionTox experiments.

### ADME information

The values of in vitro measured intrinsic hepatic clearance (CL_int_) and unbound fraction available in plasma (F_u_) were retrieved from the Integrated Chemical Environment database version 4.1 (Bell et al. 2020). Human values have been reported where available. In the case of CL_int_ when human values were unavailable, rodent values were reported. Finally, OPERA-calculated values were reported in the absence of experimental data (see Supplementary Material 5). This information enables further downstream biokinetic analyses to allow for species extrapolation and human-relevant estimations by employing physiologically based toxicokinetic modeling for reverse and forward dosimetry.

### Data visualization

The PrecisionTox Data Visualization Tool (PDVT) is a web-based tool developed as a Flash application using Cytoscape.js (Franz et al. 2016) for network visualization (https://dex.precisiontox.org/chem/). The source code is freely available at: https://github.com/precisiontox/chem-viz.

## Results

### Selection of chemicals for the PrecisionTox collection

Utilizing the protocols for chemical nomination, triaging, and purchasing presented in Fig. 1, a collection of 200 chemicals was selected from >1,500 candidates for subsequent toxicological and omics analyses (see Supplementary Material 6). As an example of the process, a list of 188 unique DNTs was assembled from information collected by the NTP, ECVAM, and ASPIS. Physicochemical and toxicological data were collected for nominees from public databases (see Supplementary Material 2). This was a semi-automated process; therefore, all types of data were collected for all nominees, independent of their use in the final chemical selection.

Some nominees were deselected based on physicochemical properties. For example, trichloroethylene has an air–water partition constant of *D*_aw_ = 0.40, which is greater than the 10^−4^ cut-off; PBDE 47 with a *K*_ow_ = 6.8 is too hydrophobic for testing in our systems (*K*_ow_ > 4). Information on availability, cost, and other shipping restrictions for the remaining candidates were then collected and used to deselect from the remaining candidates. Several of the nominees are restricted substances (e.g. cocaine, heroin) or too toxic to use under normal laboratory conditions (e.g. methylmercury). Additional nominees were removed based on the calculated amounts needed for testing in the PrecisionTox model systems and associated costs. For example, thapsigargin, domoic acid, and thalidomide would have cost €78,000, €19,000, and €1,400, respectively, making their incorporation into the collection problematic. For instance, the triaging protocol outline in Fig. 1 reduced the original 188 DNT nominees to 30 chemicals, which were included in the collection.

### Characteristics of the PrecisionTox chemical collection

The chemicals in this collection, including identifiers (CAS number, DSSTox substance ID, SMILES and InChIKey) and Toxicity Groups are presented in Supplementary Material 6. Additionally, Chemical and Products Database (CPDat) use categories are included (Dionisio et al. 2018; US Environmental Protection Agency 2025). CPDat is a database maintained by the US EPA that catalogues commercially sourced chemicals, using standard product categories, based on how they are used. The value of providing use categories is to avoid a bias by a focus on a certain group of chemicals. For example, industrial chemicals are typically not designed for biological activity. Therefore, a focus on such chemicals could limit the applicability domain of NAMs developed with a focus on such chemicals.

**Fig. 2.**
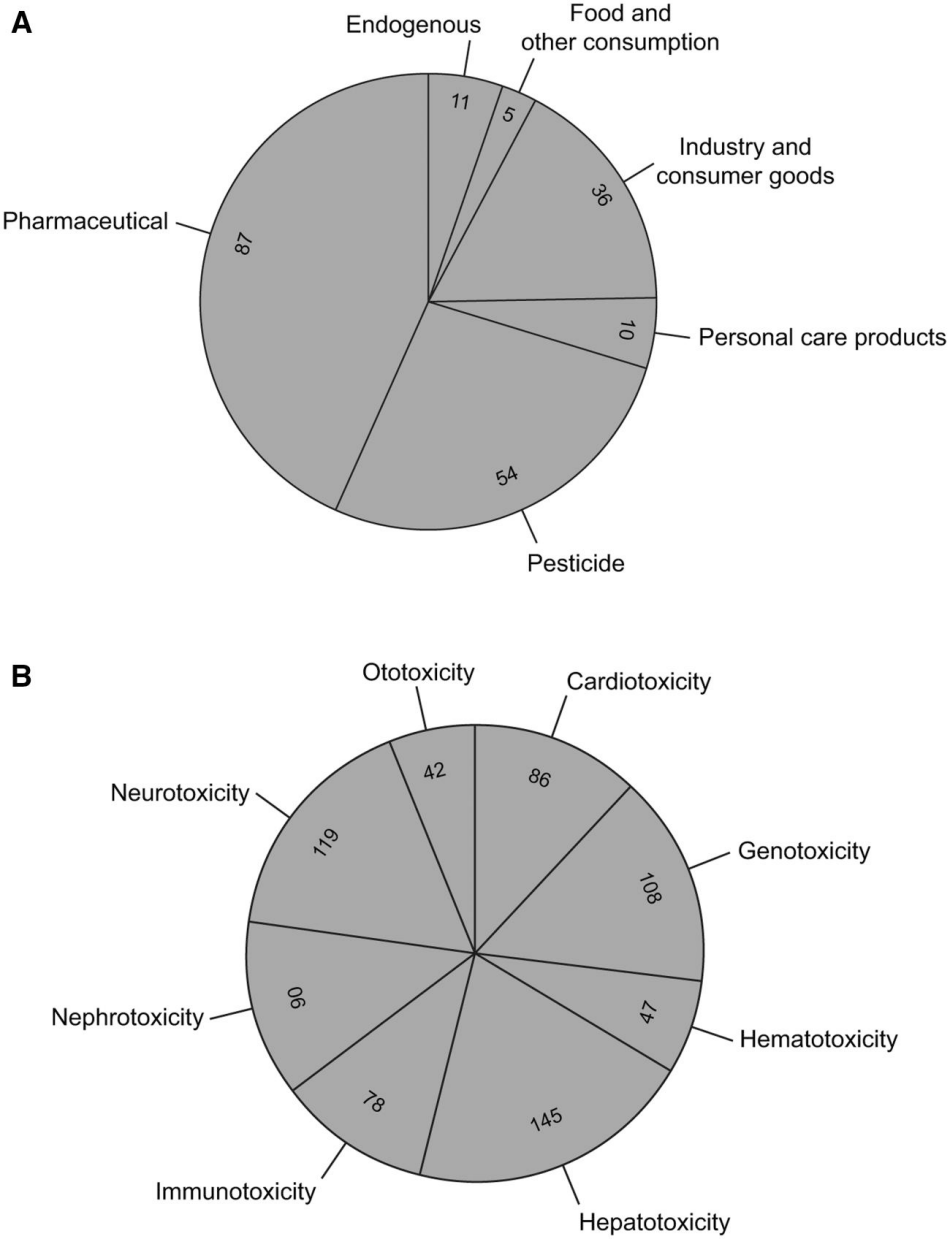
Distribution of chemicals in toxicity and use categories. Chemicals were assigned to toxicity A) or use B) categories. Values in the pie sections are the number of chemicals in that category. Use and toxicity values for each chemical can be found in Supplementary Materials 6 and 7.

Chemicals in the PrecisionTox collection are associated with 300 CPDat use categories, with the majority identified as drugs, industrial manufacturing compounds, pesticides, and food additives. Additionally, they mapped to 1 of 6 use categories that are associated with chemicals found in human plasma (Braun et al. 2024) (Fig. 2A).

#### Toxicology properties

**Fig. 3.**
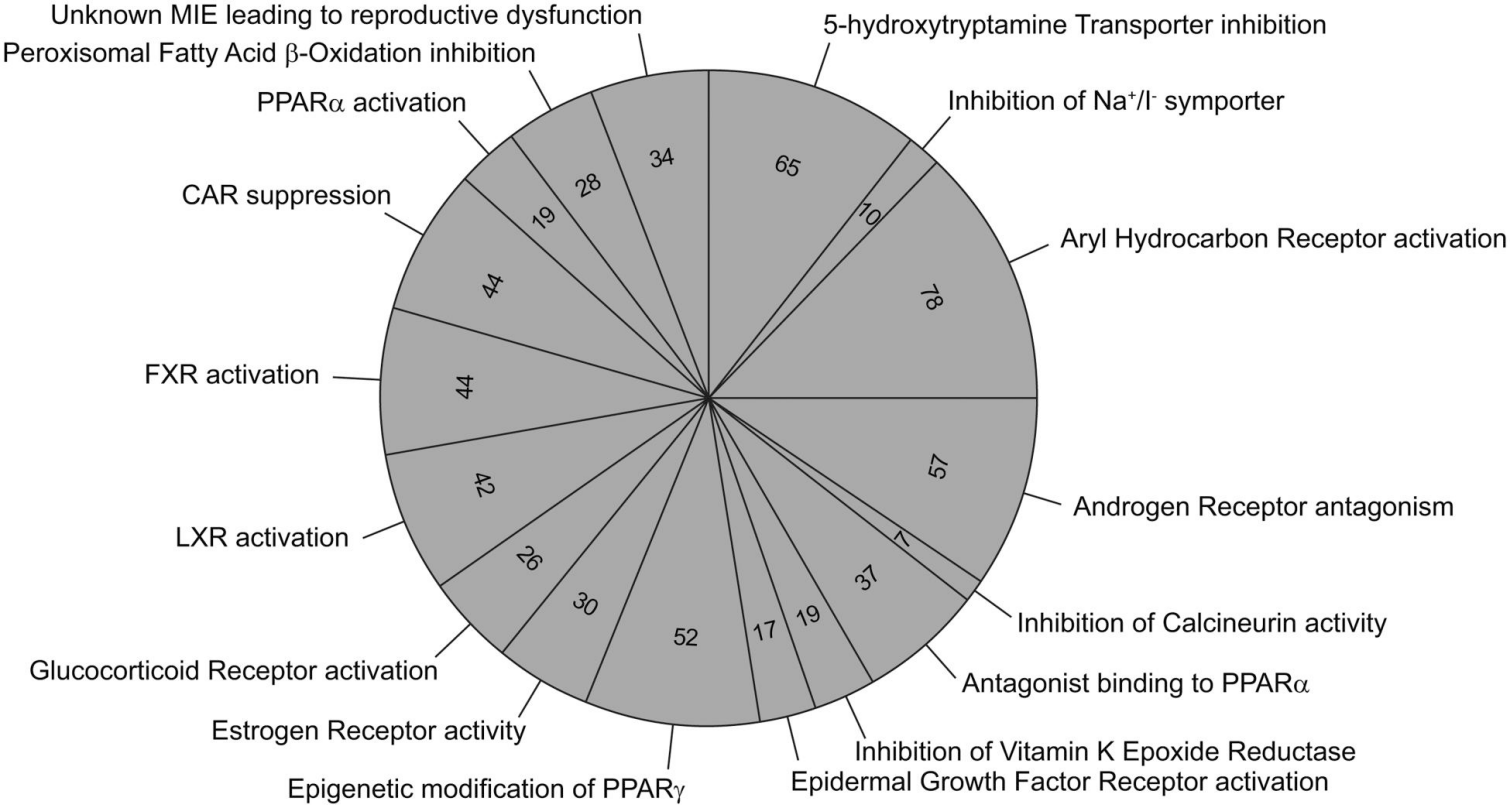
Distribution of chemicals in adverse outcome pathways. Chemicals were mapped to AOPs using the EPA AOP-DB and the number of chemicals in each AOP presented. To simplify the figure and minimize redundancy, several AOPs were combined into a single pathway. For example, 5-hydroxytryptamine transporter (5-HTT) inhibition leading to population increase (AOP ID195); 5-HTT inhibition leading to decreased shelter seeking and increased predation (AOP ID98); 5-HTT inhibition leading to population decline (AOP ID97); 5-HTT inhibition leading to decreased reproductive success and population decline (AOP ID203); and 5-HTT inhibition leading to increased reproductive success and population increase (AOP ID204) were combined to the group labeled “5-hydroxytryptamine transporter inhibition.” Values in the sections are the number of chemicals in that category. AOP information can be found in Supplementary Material 7.

Toxicological properties, including toxicity category and baseline toxicity predictions for all test animal species and Adverse Outcome Pathways (AOPs) of the chemical collection, are presented in Supplementary Material 7. AOPs, Key Events, and Molecular Initiating Events associated with each chemical were identified by searching the EPA’s Adverse Outcome Pathway Database (AOP-DB) (Mortensen et al. 2021). The AOP-DB was searched with the DTXSIDs of the PrecisionTox chemicals. Of the 200 chemicals in the collection, all except 24 are assigned to 1 or more of 8 toxicity categories (Fig. 2B). The majority of the chemicals are assigned to multiple categories, with hepatotoxicants and neurotoxicants being the most populated. Of the 200 chemicals, 87 were associated with 1 or more unique AOPs (Fig. 3) (see Supplementary Material 7).

#### Baseline toxicity predictions

**Fig. 4.**
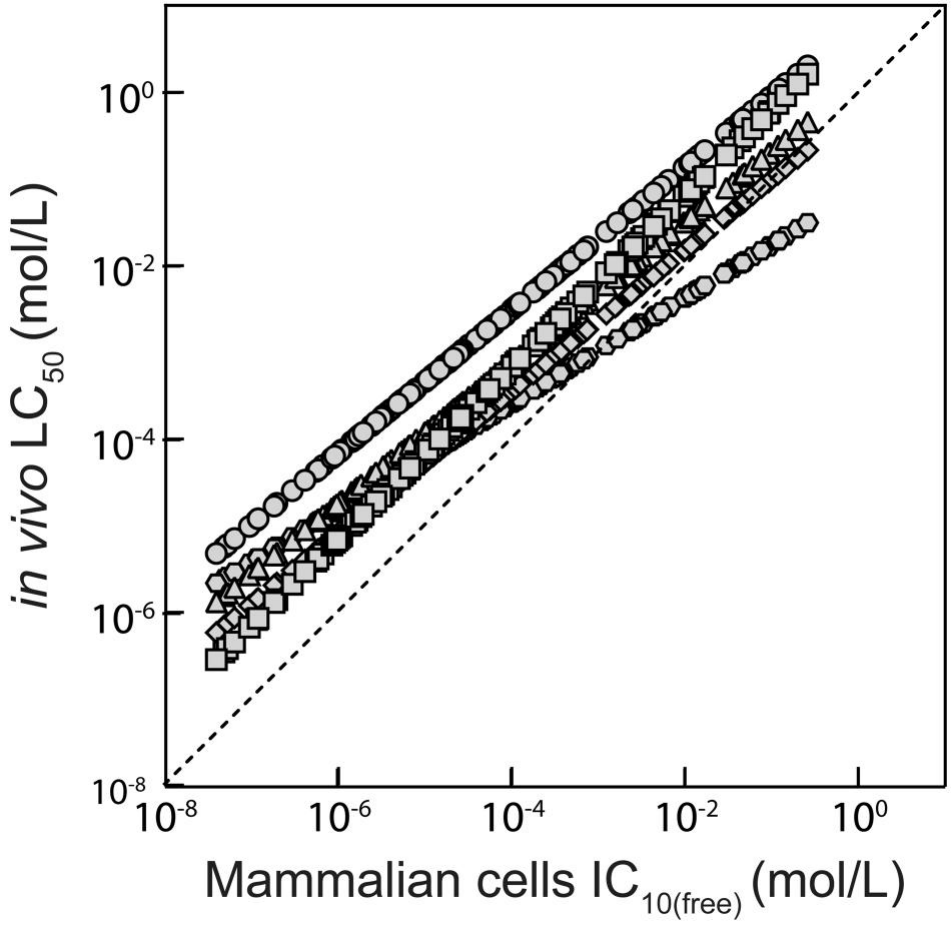
In vitro–in vivo comparison between the baseline toxicity predictions for mammalian cell cytotoxicity IC_10(free)_ normalized to freely dissolved concentrations (in vitro) and in vivo LC_50_ values for *D. melanogaster* (circle), *D. rerio* (square), *C. elegans* (triangle), *D. magna* (diamond), and *X. laevis* (hexagon).

The predicted baseline toxicity represents the minimal toxicity associated with any chemical (Qin et al. 2024). It can be used for planning of the dosing concentrations as well as the interpretation of the data. The specificity ratio, the ratio of the predicted IC_10_ or LC_50_ to the corresponding experimental data, indicates which chemicals are toxic because they are hydrophobic and have a high baseline toxicity or if they are reactive or have specific targets. QSAR predictions for the PrecisionTox in vivo models varied within a factor of 100 per chemical (Fig. 4; see Supplementary Material 7). The predicted in vitro IC_10(free)_ was similar to the predicted in vivo LC_50_. In general, hydrophobic chemicals were less sensitive in vivo, and hydrophilic chemicals have a similar sensitivity.

#### ADME information

To provide additional information on the chemical collection, potential hepatic clearance rates were calculated. Results show intrinsic hepatic clearance values above 10 μl/min/10^6^ cells for 75 compounds. Four substances have rapid clearance values above 100 μl/min/10^6^ cells, as determined by human in vitro assays: Propofol (CAS No. 2078-54-8), methylparaben (CAS No. 99-76-3), bisphenol E (CAS No. 2081-08-5), and chlorpromazine (CAS No. 69-09-0), with clearance rates of 107, 107.5, 119, and 195.1 μl/min/10^6^ cells, respectively. Based on experimental in vitro human and rodent data, approximately one-third of the chemicals in the PrecisionTox collection have clearance values below 2.5 μl/min/10^6^ cells. This observation indicates a very slow rate of metabolism in mammalian test systems. An IA clearance value of 0 indicates little to no metabolism, a potential for bioaccumulation, or a lack of data. This highlights the need for a thorough review of existing data and potentially additional testing to better understand the metabolic fate of these chemicals (Gouliarmou et al. 2018). Human plasma protein-unbound fraction values for 133 compounds were ≥50%, indicating a high availability of the free chemical in plasma. This could lead to an increased potential for interaction with molecular targets, including enzymes or receptors. Additionally, metals and inorganic compounds are out of the model’s applicability domain, and data for tunicamycin, cadmium chloride, sodium arsenite, tributyltin, triethyltin bromide, sodium perchlorate, and lithium (cation) were unavailable (see Supplementary Material 5).

#### Physicochemical properties

**Fig. 5.**
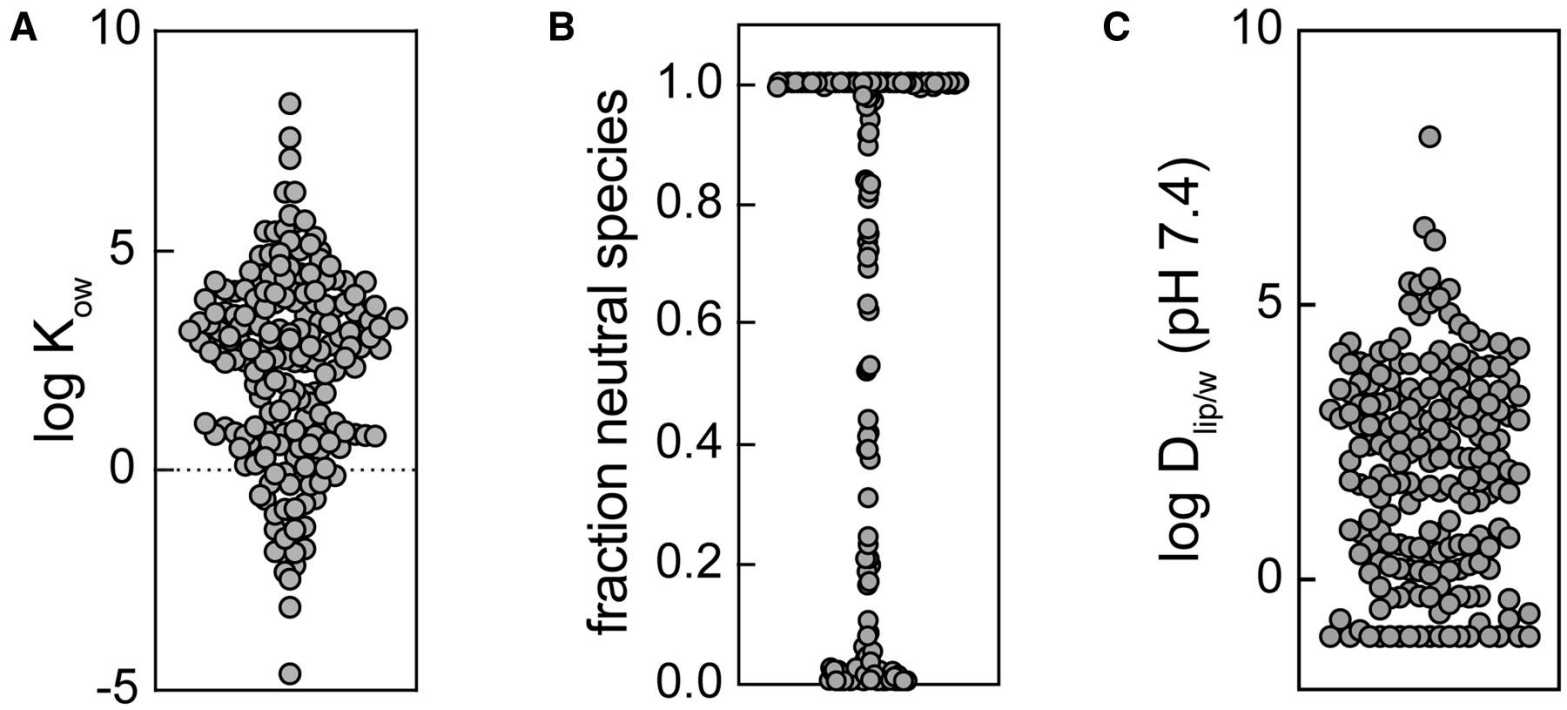
Physicochemical properties of the PrecisionTox chemical collection. A) Distribution of octanol–water partition constants *K*_ow_ of the neutral species of all test chemicals. B) Distribution of fractions of neutral species of all test chemicals derived from the acidity constants pKa. C) Distribution of the ionization-corrected liposome water distribution ratio (*Dlip/w*) of all species at a pH 7.4. Detailed data can be found in Supplementary Material 4.

The *K*_ow_ of the neutral species range from −4.63 to 8.50, covering 12 orders of magnitude with a mean of 2.31 (Fig. 5A). Over 80 of the chemicals are 75% to 100% in neutral form, almost 60 are more than 80% charged, with the remainder having multiple charges at pH 7.4 (Fig. 5B). The *D*_lip/w_ (pH 7.4) of the collection ranges from −1 to 8.5, with more than 80% of the compounds between −1 and 4 (Fig. 5C). The lowest value was set to −1, because according to Gobas et al. (1988), that is the empirical lowest distribution ratio. Additionally, the QSAR is not valid if the affinity to membrane bilayers is too low. We also predicted binding to plasma (albumin) and SPs. As most were derived from the log *K*_ow_ or *D*_lip/w_, the distributions look similar to Fig. 5C (see Supplementary Material 4).

#### In vitro distribution modeling to estimate availability

**Fig. 6.**
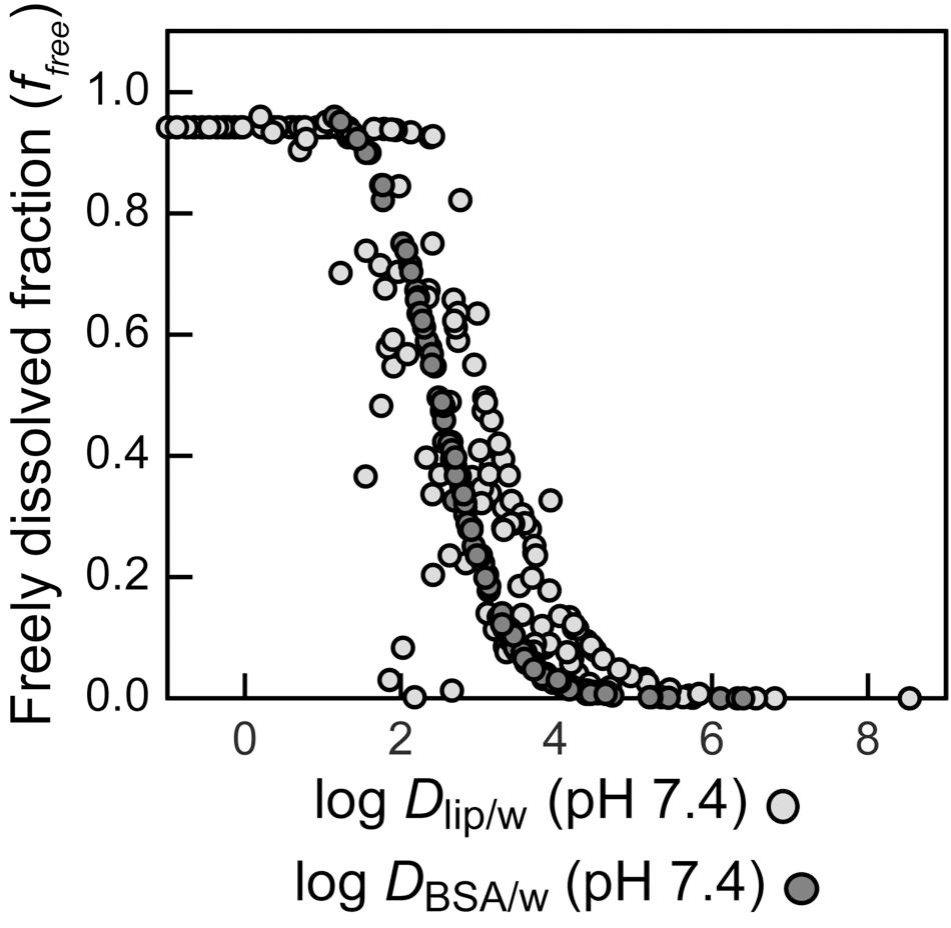
Freely dissolved fraction *f*_free_ in the bioassay medium (Equation 1) as a function of liposome–water distribution ratio (*D*_lip/w_ [pH 7.4]) (light gray dots) or protein–water distribution ratio (*D*_BSA/w_ [pH 7.4]) (dark gray dots).

The fraction of freely dissolved chemical in the bioassay medium (*f*_free_*)* decreases with increasing hydrophobicity (Fig. 6). Chemicals with log *D*_lip/w_ <3 are typically fully dissolved, and those with log*D*_lip/w_ >5 are almost completely bound. Fulvestrant (CAS No. 129453-61-8) is extremely hydrophobic, log*D*_lip/w_ >8, which is outside the domain of the selection criteria, so the prediction should be treated with caution.

As the serum protein binding dominates the reduction of *f*_free_, the relationship between *f*_free_ and *D*_BSA/w_ is much more uniform than the relationship between *f*_free_ and *D*_lip/w_ (Fig. 6). This is because log*D*_lip/w_ and *D*_BSA/w_ are only proportional for neutral and positively charged chemicals, but anions bind stronger to proteins than to lipids.

In a medium that is supplemented with 10% FBS, the fraction taken up into the cells is negligible, and as the freely dissolved concentrations are low for the very hydrophobic chemicals, the binding to the plastic plates is also negligible. In addition, many test chemicals are partially or fully charged, and charged chemicals do not diffuse into the polymers. However, many cell culture plates are treated to make cells better adherent, and the tissue culture treatment makes the plastic surfaces anionic, allowing cations to bind. Unfortunately, there are no binding constants available yet for treated plate materials.

Uptake into cells and zebrafish embryos can be predicted reliably using established models (Grasse et al. 2024; Proença et al. 2021), but for all other assays, such prediction models do not exist because baseline toxicity QSARs are based only on empirical correlations with *D*_lip/w_ and not on a mechanistic disposition model.

### PrecisionTox data visualization tool

The PDVT is a web-based application developed to facilitate the exploration and analysis of the PrecisionTox chemical collection: https://dex.precisiontox.org/chem. Currently, the database contains all of the physicochemical and toxicological data reported in this manuscript and supplemental materials. The database and PDVT are open-ended, and in the future will include novel experimental data generated by PrecisionTox.

**Fig. 7.**
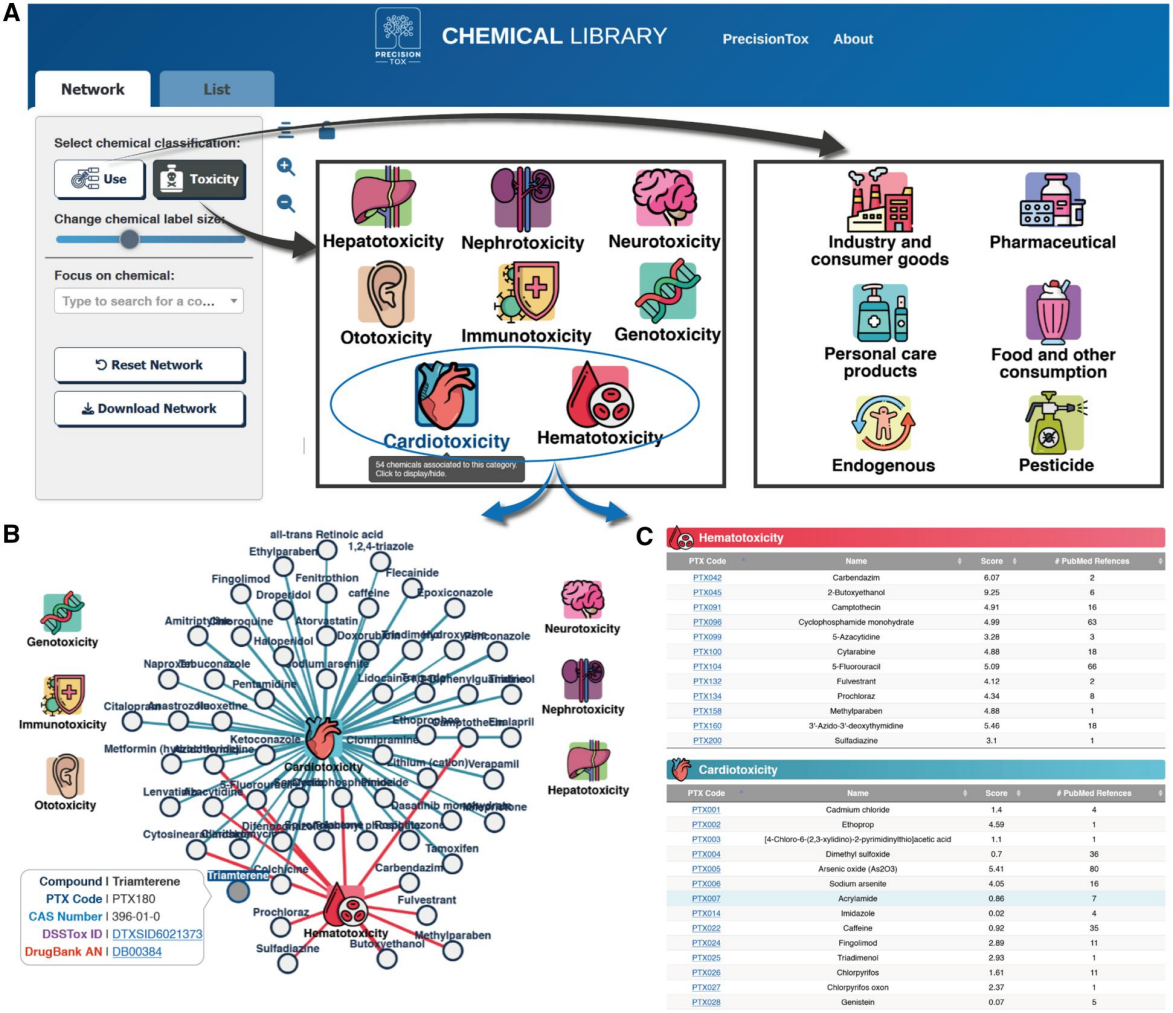
General view on PDVT. A) An interactive diagram shows the different categories for chemical classification according to 2 different classification systems: “use” and “toxicity endpoint,” which the user can toggle between. B) Clicking on 1 or more of these nodes will display all the chemicals included in those categories as additional child nodes. Chemical can be associated to more than 1 category, which is represented by differently colored edges. C) In addition to the network, dynamically changing table will show the full list of chemicals associated to the last clicked category (https://dex.precisiontox.org/chem).

The PDVT features an interactive network that allows users to visualize how chemicals are classified according to 2 main categories: Use and Toxicity Endpoint (Fig. 2). PDVT has the ability to toggle between these classification systems. The differently colored nodes represent specific subcategories within each classification. For example, under the Use classification, nodes include *pesticide*, *pharmaceutical*, *industry*, *consumer goods*, and others (Fig. 7A). Each node can be clicked to reveal all chemicals associated with that particular subcategory, which will be shown connected to it via edges (Fig. 7B). These chemicals are simultaneously displayed in a table below the graph, providing a clear and organized view of the corresponding entries (Fig. 7C).

**Fig. 8.**
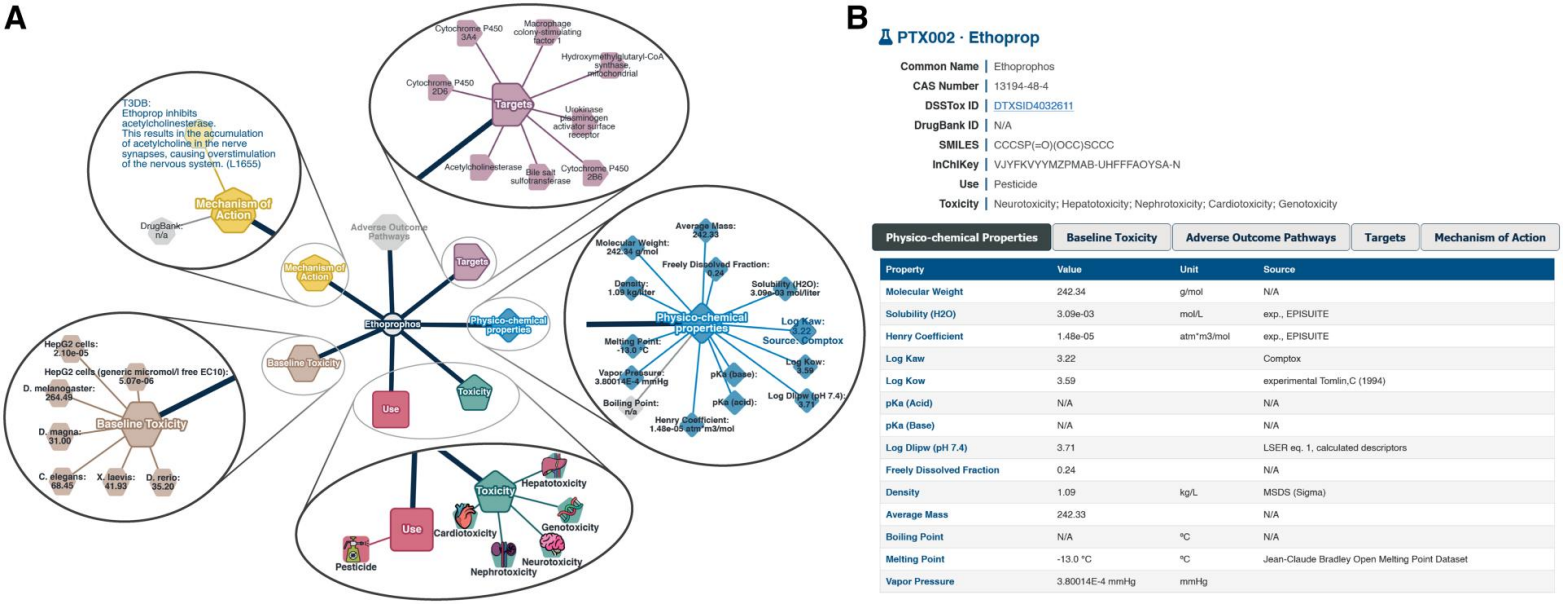
Single chemical view on PDVT. A) The network shows the selected chemical surrounded by nodes that represent the collected chemical information categories. Each can be expanded individually by clicking to show additional nodes displaying the associated data. B) All this information is also displayed below in a more typical knowledge base format including the diverse chemical identifiers and a table where users can toggle between the different types of annotations.

The web application has a search functionality that allows users to focus on individual chemicals. The single chemical view page comprises a new network graph that displays all collected information for that compound. This includes its use and toxic endpoint categories, physicochemical properties, mechanisms of action, baseline toxicity data, molecular targets, and adverse outcome pathways. Each of these main attributes is represented as a node that can be clicked to display all specific data as additional child nodes (Fig. 8A). For example, physicochemical properties comprise up to 14 different attributes, including molecular weight, density, Henry coefficient, etc., whereas mechanisms of action contain relevant available annotations obtained from DrugBank and T3DB. For further convenience, chemical information is also displayed in a structured table format beneath the graph (Fig. 8B). A snapshot of the current network view can be obtained at any time in image or vector graphics formats. Finally, the web application includes a comprehensive table listing the entire chemical collection. This table consolidates all available data for each chemical into clearly defined columns, encompassing physicochemical properties, use, toxicity classifications, mechanisms of action, and other relevant information. Users can filter and sort the table as needed to support targeted analysis, as well as download it in different formats.

## Discussion

Over the past several decades, the ability to screen large numbers of chemicals for their biological effects has become an integral part of the discovery of new drugs, pesticides, and other commercial products, as well as toxicity testing. Additionally, HTS and associated data analysis workflows have contributed to the development of novel test methods. This has been accomplished by assessing their biological relevance via the interrogation of large numbers of chemicals to identify applicability domains and potential limitations, such as chemical space coverage and/or the range of expected responses. High-throughput screens also assess the reliability of a test method to increase reproducibility and minimize variability.

The starting point for the utilization of any HTS is the creation of a chemical library. There are 2 basic components in the creation of a chemical library. First is to define the parameters or chemical space of the collection: Targets for a specific protein/receptor, pathway/mechanism of response, disease/health endpoint, or to test a variety of responses (Richard et al. 2021). Multiple approaches have been used to create chemical libraries, which include synthesizing congeners of biologically active compounds (Sahoo et al. 2022), designing virtual libraries based on QSAR (Nowak et al. 2024), collecting chemicals with known responses (Sund and Deceuninck 2021), and pooling compounds of interest selected by various parties (Attene-Ramos et al. 2013). The second component in the creation of a chemical library are logistic and practical considerations: Can a compound be purchased or synthesized in the required amount, at the necessary purity, and at an acceptable cost? Also, can the chemical be tested in the selected biological system under available laboratory conditions?

Here, we report on the creation of a unique, fit-for-purpose chemical library designed to test the phylotoxicology hypotheses (Colbourne et al. 2022; PrecisionTox Consortium 2023). In contrast to other libraries, the PrecisionTox chemical collection was not created to target a specific disease, protein, or pathway. Rather, diversity was at the center of its design. To select and subsequently test this chemical collection, a robust chemical identification and logistics protocol was developed (Fig. 1). From >1,500 nominees, 200 compounds were selected, purchased, and distributed to PrecisionTox testing laboratories in the EU, the United Kingdom, and the United States. Nominations were collected to reflect compounds with a variety of potential MoA. Problematic nominees were removed based on physicochemical properties obtained from public databases or calculated de novo. Practical considerations (cost, purity, availability, and the ability to send the compounds to the international PrecisionTox testing laboratories) were also important deciding factors. Ultimately, diverse representation throughout chemical space determined a nominee’s inclusion in the collection.

With a few exceptions, the protocol functioned as anticipated. One problem occurred due to an unforeseen chemical property. N-(hydroxymethyl)acrylamide (CAS No. 924-42-5) unexpectedly oligomerized in solution and therefore was removed from testing. Logistical challenges related to regulatory restrictions affected the inclusion of pentobarbital (CAS No. 76-74-4), which is subject to export prohibitions due to its potential use in certain jurisdictions. As a result, it was also removed from the collection to comply with applicable laws and regulations.

Based on the selection criteria, the final PrecisionTox collection is diverse, but due to the limited number of chemicals that could be tested, it is not inclusive. Chemicals in the collection target several of the organs typically associated with human toxicity, including the liver, kidney, heart, and the nervous and immune systems (Table 1, Fig. 2B). Chemicals that target the lung, skin, and the visual apparatus were excluded during selection. As each of these organ targets are not present in several or all of the PrecisionTox model species, they were not suitable for testing the phylotoxicology hypothesis.

This chemical collection is biased toward pharmaceuticals and pesticides (Fig. 2A). This is consistent with the nomination process and selection protocol, as large amounts of physicochemical, toxicological, and exposure data are available for chemicals in these categories. These use categories typically comprise compounds with a specific MoA, which make them suitable as reference compounds. These reference compounds can be used to define MoA, mechanism of toxicity, biomarkers, and ethology for chemicals. In contrast, non-specifically acting compounds (e.g. with toxicity driven by hydrophobic interaction with cellular membranes) would result in effect concentration close to QSARs predictions (e.g. Huchthausen et al. 2023, 2024) and similar effect concentrations across phylogenetically distant groups. Hence, for the development of alternative approaches for the screening of chemicals based on NAMs and reduced reliance on animal tests (Sewell et al. 2024), particularly methods capturing specific modes of interactions (e.g. binding to specific target molecules) are needed. This is also a prerequisite to use the biological effect patterns in a read-across approach beyond the similarity of chemical structure (Escher et al. 2019b).

The retrieval of information on the physicochemical properties and baseline toxicity predictions helped the testing laboratories handle such a diverse set of chemicals and dose them appropriately. A large number of the chemicals have a specific MoA. Three compounds that were expected to show no specific MoA were intentionally included as reference compounds to demonstrate that experimental effect concentrations for these compounds would be close to QSAR predictions. It can be expected that, specific to the in vivo model, more compounds will exhibit effect concentrations close to QSAR prediction, e.g. when the specific target may not be available in the corresponding model. Additionally, compounds included due to their environmental occurrence and to obtain missing toxicity information also may represent non-specifically acting compounds. By comparing the experimentally observed effects (i.e. cytotoxicity, mortality, mechanism-specific endpoints) to the predicted baseline toxicity, it is possible to quantify and estimate the reliably to detect specific effects of NAMs and benchmark them with respect to traditional animal-test-based approaches.

It should be noted that compounds with difficult physicochemical properties (volatility, hydrophobicity) were excluded for practical reasons. Such compounds require special exposure regimes. Excluding such compounds increases the throughput for benchmarking the approach(es) targeted with our library. Given the availability of special testing routines for difficult compounds, however, this does not limit per se the application domain for these compounds. Testing of volatile or hydrophobic chemicals is a regulatory requirement (see OECD 2019). Testing these compound in the context of PrecisionTox, however, would require additional efforts that would reduce the number of compounds included in the chemical collection to develop NAMs. It is therefore advisable to avoid these compounds during NAM development. Using appropriate guidance on testing difficult compounds, they can, however, be tested using developed NAMs.

One issue that needs to be taken into account when building a chemical library is the amount of available data for any individual compound. Experimental and theoretical data are readily available on the physicochemical properties through PubChem, CompTox, and other databases (see Supplementary Material 2). Where data are missing from public databases, de novo calculations can be used to fill these gaps. All of the chemical nominees for this collection had sufficient physicochemical data for triaging. Additionally, the selected compounds are also readily available at high purities, low cost, and international shipping regulations are well defined.

In vivo and in vitro toxicity, AOP, exposure, and toxicity mechanism data are generally not as complete. For example, of the 200 chemicals in the PrecisionTox collection, only 87 have been assigned to an AOP; less than half of the collection are found in DrugBank, and <50 have human exposome information. This paucity of toxicological information reflects the need for additional data and justifies its inclusion in this multipurpose library for toxicity testing. The ADME in vitro parameters, CL_int_ and F_u_ (human and rat), were extracted from the Integrated Chemical Environment database only after the chemical selection process had been completed, with the purpose of enabling further downstream analysis, such as PBK modeling to estimate effective dose-extrapolation assumptions to humans.

To summarize, from over 1,500 nominees, a collection of 200 chemicals was obtained for the PrecisionTox Consortium with diversity in chemical space as its core principle. This collection is a well-characterized set of compounds that will be used to test the phylotoxicology hypothesis. Selections are based on physicochemical and toxicological properties, as well as practical considerations. This report highlights the need for strategic selection criteria for any chemical library development, ensuring the appropriate level of coverage within chemical space while balancing practical constraints.

## Supplementary Material

kfaf126_Supplementary_Data
