# Supplementary material for: The PrecisionTox chemical library: creation of a chemical collection to discover evolutionary conserved biomolecular signatures of toxicity
Source: Toxicol Sci. 2025 Nov 4;208(2):317–29. doi: 10.1093/toxsci/kfaf126 (PMC12646593; doi:10.1093/toxsci/kfaf126)
Supplement: kfaf126_Supplementary_Data [file kfaf126_supplementary_data.zip › kfaf126_Supplementary_Data/toxsci-25-0240-File011.docx]

| **Supplemental Table 1. Databases used in Chemical Selection for the PrecisionTox Collection** | | | |
| --- | --- | --- | --- |
| **Database** | **Link (June 2024)** | **Reference** |  |
| CompTox Chemicals Dashboard | https://www.epa.gov/comptox-tools | (Williams *et al.*, 2021) |  |
| Adverse Outcome Pathway | https://aopdb.epa.gov/ | (Mortensen *et al.*, 2021) |  |
| DrugBank | https://go.drugbank.com/ | (Wishart *et al.*, 2018) |  |
| Integrated Chemical Environment | https://ice.ntp.niehs.nih.gov/ | (Bell *et al.*, 2017) |  |
| Comparative Toxicogenomics | https://ctdbase.org/ | (Davis *et al.*, 2023) |  |
| ToxRefDB | https://cfpub.epa.gov/ | (Feshuk *et al.*, 2023) |  |
| Toxin and Toxin Target | http://www.t3db.ca/ | (Lim *et al.*, 2010) |  |
| Exposome-Explorer | http://exposome-explorer.iarc.fr/ | (Neveu *et al.*, 2020) |  |
| Chemical and Products Database | https://www.epa.gov/chemical-research/chemical-and-products-database-cpdat | (Dionisio *et al.*, 2018) |  |
| NeurotoxKB | https://cb.imsc.res.in/neurotoxkb/ | (Ravichandran *et al.*, 2021) |  |

Bell, S. M., Phillips, J., Sedykh, A., Tandon, A., Sprankle, C., Morefield, S. Q., Shapiro, A., Allen, D., Shah, R., Maull, E. A.*, et al.* (2017). An Integrated Chemical Environment to Support 21st-Century Toxicology. *Environ Health Perspect* **125**(5), 054501.

Davis, A. P., Wiegers, T. C., Johnson, R. J., Sciaky, D., Wiegers, J., and Mattingly, C. J. (2023). Comparative Toxicogenomics Database (CTD): update 2023. *Nucleic Acids Res* **51**(D1), D1257-d1262.

Dionisio, K. L., Phillips, K., Price, P. S., Grulke, C. M., Williams, A., Biryol, D., Hong, T., and Isaacs, K. K. (2018). The Chemical and Products Database, a resource for exposure-relevant data on chemicals in consumer products. *Sci Data* **5**, 180125.

Feshuk, M., Kolaczkowski, L., Watford, S., and Paul Friedman, K. (2023). ToxRefDB v2.1: update to curated in vivo study data in the Toxicity Reference Database. *Front Toxicol* **5**, 1260305.

Lim, E., Pon, A., Djoumbou, Y., Knox, C., Shrivastava, S., Guo, A. C., Neveu, V., and Wishart, D. S. (2010). T3DB: a comprehensively annotated database of common toxins and their targets. *Nucleic Acids Res* **38**(Database issue), D781-6.

Mortensen, H. M., Senn, J., Levey, T., Langley, P., and Williams, A. J. (2021). The 2021 update of the EPA's adverse outcome pathway database. *Sci Data* **8**(1), 169.

Neveu, V., Nicolas, G., Salek, R. M., Wishart, D. S., and Scalbert, A. (2020). Exposome-Explorer 2.0: an update incorporating candidate dietary biomarkers and dietary associations with cancer risk. *Nucleic Acids Res* **48**(D1), D908-D912.

Ravichandran, J., Karthikeyan, B. S., Singla, P., Aparna, S. R., and Samal, A. (2021). NeurotoxKb 1.0: Compilation, curation and exploration of a knowledgebase of environmental neurotoxicants specific to mammals. *Chemosphere* **278**, 130387.

Williams, A. J., Lambert, J. C., Thayer, K., and Dorne, J. C. M. (2021). Sourcing data on chemical properties and hazard data from the US-EPA CompTox Chemicals Dashboard: A practical guide for human risk assessment. *Environ Int* **154**, 106566.

Wishart, D. S., Feunang, Y. D., Guo, A. C., Lo, E. J., Marcu, A., Grant, J. R., Sajed, T., Johnson, D., Li, C., Sayeeda, Z.*, et al.* (2018). DrugBank 5.0: a major update to the DrugBank database for 2018. *Nucleic Acids Res* **46**(D1), D1074-d1082.
