# Supplementary material for: The PrecisionTox chemical library: creation of a chemical collection to discover evolutionary conserved biomolecular signatures of toxicity
Source: Toxicol Sci. 2025 Nov 4;208(2):317–29. doi: 10.1093/toxsci/kfaf126 (PMC12646593; doi:10.1093/toxsci/kfaf126)
Supplement: kfaf126_Supplementary_Data [file kfaf126_supplementary_data.zip › kfaf126_Supplementary_Data/toxsci-25-0240-File012.docx]

## Physicochemical properties.

Henry coefficients *K*_H_ were estimated using OPERA version 2.6 or were obtained from the CompTox Chemicals Dashboard (US EPA, 2023; Williams *et al.*, 2021). They were then converted to air-water partition constants *K*_aw_ using equation 1, where R is the ideal gas constant and T the temperature (25 °C).


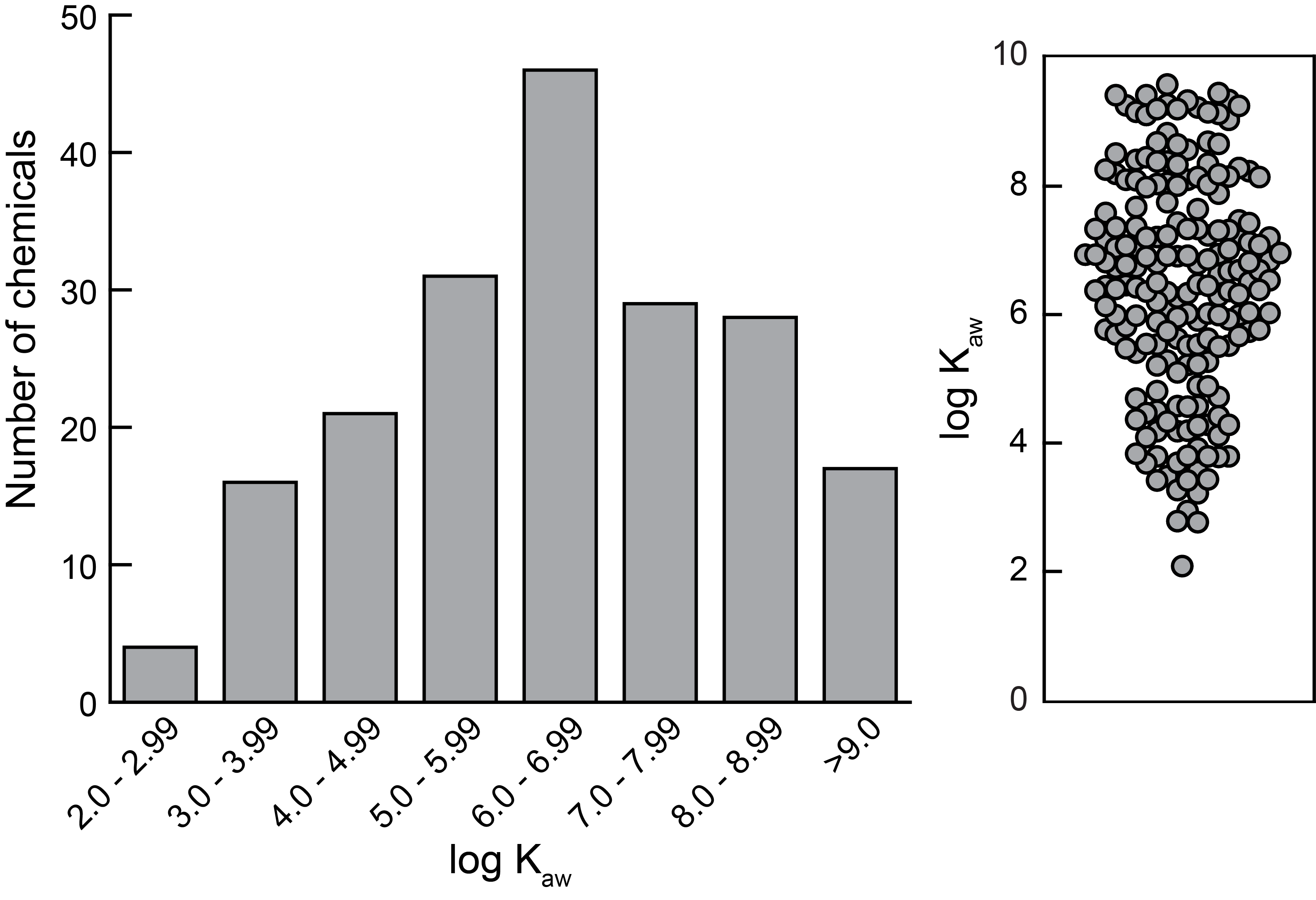


$\text{K}_{\text{aw}}\text{=}\frac{\text{K}_{\text{H}}}{\text{RT}}$ (1)

For partially charged chemicals, the *K*_aw_ was corrected for ionization to obtain the *D*_aw_(pH7.4) considering that charged chemicals do not partition to the air.

$\text{D}_{\text{aw}}\text{(pH7.4)}\text{=}{\text{f}_{\text{neutral}}\text{K}}_{\text{aw}}$ (2)

The fraction of neutral species *f*_neutral_ was calculated from the acidity constants p*K*_a_. Experimental acidity constants p*K*_a_ were taken from literature or measured for most of the chemicals using a Sirius T3 titrator (Huchthausen *et al.*, 2024; Niu *et al.*, 2022). If no experimental data were available or the prediction indicated that speciation was irrelevant because the chemical did not have any ionizable functional groups, the p*K*_a_ values were predicted with ACD pKa/GALAS (ACD/Percepta, 2015). The fractions of all species i, *f*_i_ (neutral or zwitterionic [= net neutral] *f*_neutral_, negative, double negative, positive, double positive) were calculated with the Henderson Hasselbalch equation, which was derived for complex multiprotic substances as reported previously (Escher *et al.*, 2020).


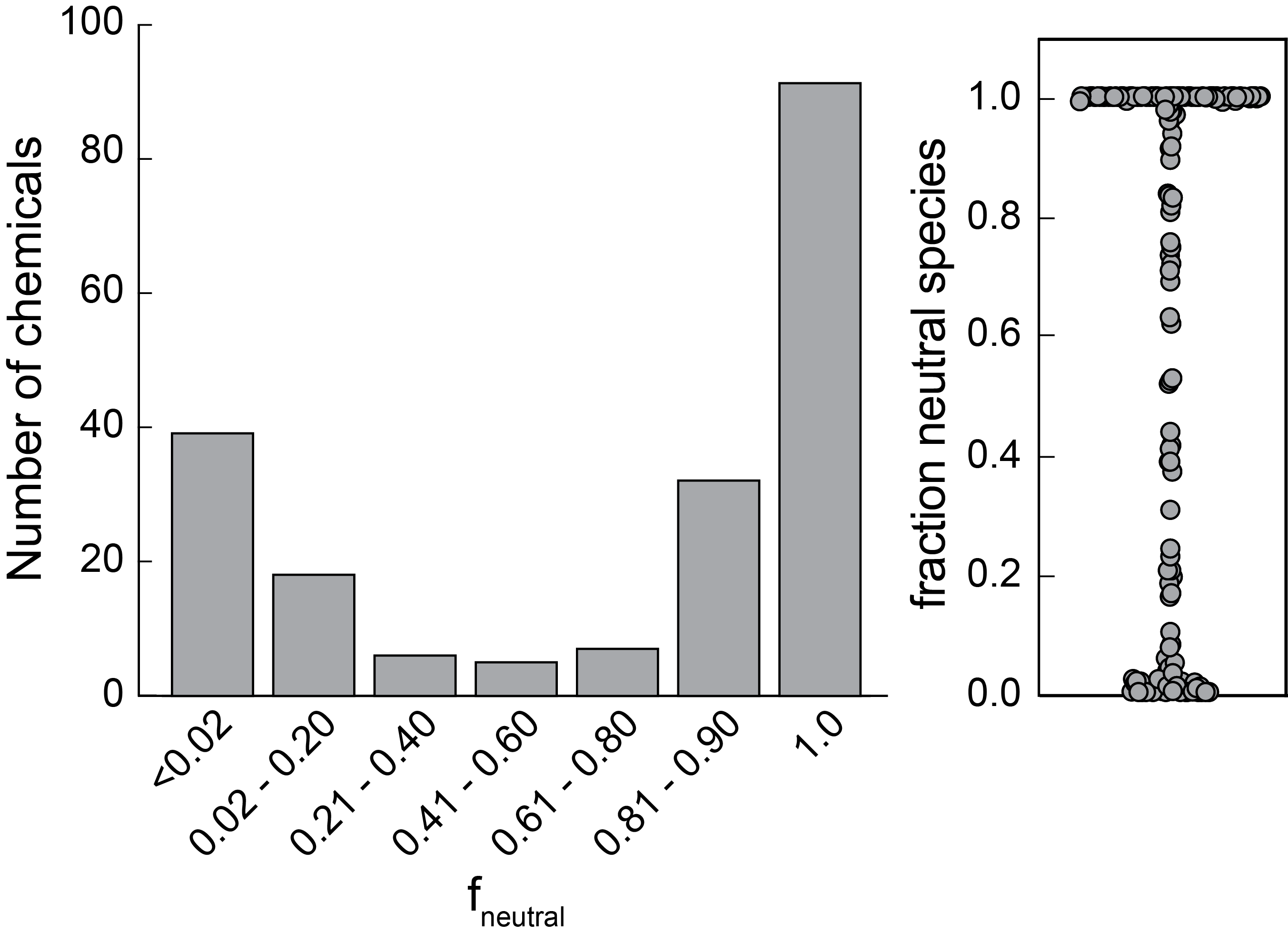


Octanol-water partition constants *K*_ow_ were preferentially experimental values retrieved from original papers or the CompTox Chemicals Dashboard. If no experimental data were available, the *K*_ow_ were predicted with a poly-parameter linear solvation energy relationship (LSER) using the UFZ-LSER database (Ulrich *et al.*, 2021). If the input parameters for the model were not available, the results from two prediction methods (KOWWIN v1.67, ACD/Labs Consensus) were downloaded from CompTox Chemicals Dashboard. As we noted earlier (Adamovsky *et al.*, 2024), OPERA predictions were very inconsistent and therefore these values were not considered and only the means of the prediction by KOWWIN v1.67 and ACD/Labs Consensus were used.


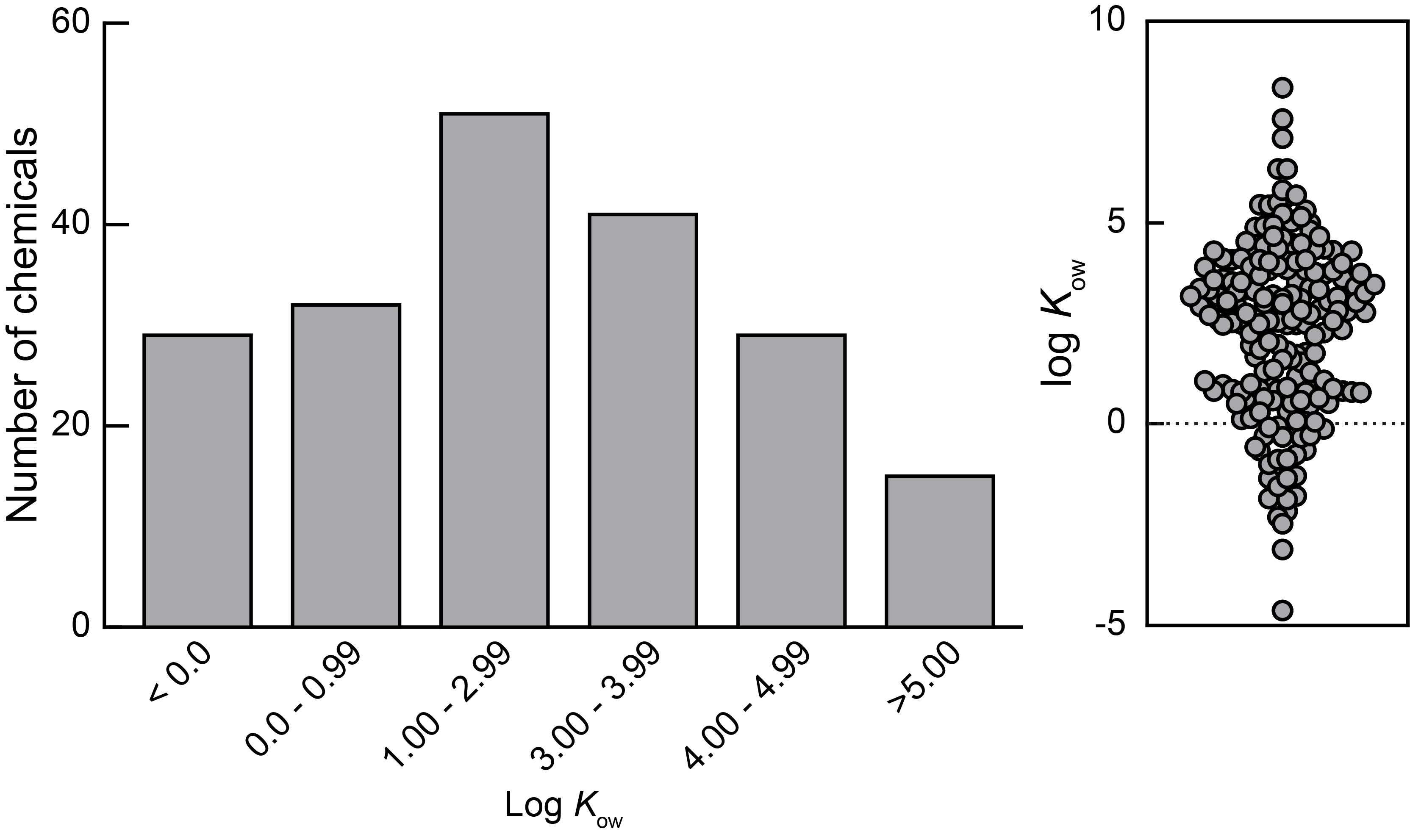


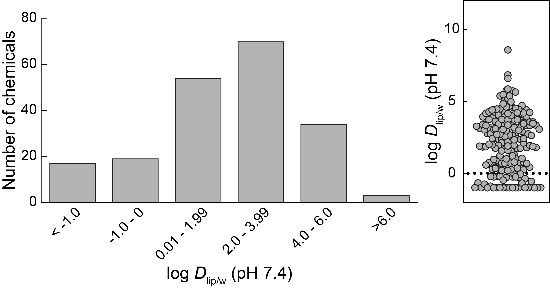
The *K*_ow_ was used to predict the liposome-water partition constant (*K*_lip/w_) of the neutral species unless experimental *K*_lip/w_ were available in literature. The *K*_lip/w_ refers to partition between water and lipid membrane bilayer vesicles, so-called liposomes, used as surrogate for membrane lipids. It should be noted that the ionization corrected *K*_ow_ was not used because binding of charged species to membranes and proteins is not negligible (Escher *et al.*, 2004) and the use of *D*_ow_(pH) leads to erroneous predictions of membrane affinity and bioaccumulation of ionizable chemicals (Escher, et al., 2020).

If no experimental data were available, the *K*_lip/w_ of the neutral species were calculated with a LSER (Ulrich, et al., 2021), and if no LSER descriptors were available, the log *K*_lip/w_ were predicted with a linear regression from log *K*_ow_ (Endo *et al.*, 2011a) (eq. 3)

${\text{log}\text{K}}_{\text{lip/w}}\text{=}\text{1.01}\text{×}\text{log}\text{K}_{\text{ow}}+0.12$ (3)

Then for ionizable chemicals, the liposome water distribution ratio (*D*_lip/w_) of all species at a given pH (here: 7.4) was either retrieved form experimental literature data or calculated from the fractions of all species in the water phase *f*_i_. The liposome-water partition constants (*K*_lip/w_) of all species i and *f*_i_ were combined to derive the *D*_lip/w_(pH) of all species (eq 4).

$\text{D}_{\text{lip/w}}\left( \text{pH} \right)\text{=}\frac{\sum_{\text{i=1}}^{\text{n}} \text{C}_{\text{lip}}\text{(i}\text{)}}{\sum_{\text{i=1}}^{\text{n}} \text{C}_{\text{w}}\text{(i}\text{)}}\text{=}\sum_{\text{i=1}}^{\text{n}} \text{f}_{\text{i}}\text{×}\text{K}_{\text{lip/w}}\text{(i)}$ (4)

Again, experimental data were preferred if available for pH of 7 to 7.4, otherwise the *D*_lip/w_ was calculated with a simplified equation 5. This was done because there are no prediction models for other than neutral species and in practice the *K*_lip/w_ of charged chemicals is often about one to two orders of magnitude lower than that of the corresponding neutral species (Escher, et al., 2020).

$\text{D}_{\text{lip/w}}\left( \text{pH} \right)\text{=}\text{f}_{\text{neutral}}\text{×}\text{K}_{\text{lip/w}}\text{(neutral)}\text{ + (1- }\text{f}_{\text{neutral}}\text{)}\text{×}\frac{\text{K}_{\text{lip/w}}\text{(neutral)}}{10}$ (5)

Bovine serum albumin (BSA) serves as surrogate for protein binding in medium (*K*_BSA/w_). If experimental data were available, the *D*_BSA/w_ measured at pH 7 to 7.4 were preferred. If no experimental data were available, the *K*_BSA/w_ of the neutral species was calculated with a LSER as was also done for *K*_ow_ (Ulrich, et al., 2021). Alternatively, the *K*_BSA/w_ was predicted from log *K*_ow_ (Endo *et al.*, 2011b). The *K*_BSA/w_ was assumed to be the same as for neutral chemicals. Anions have a higher affinity to BSA. Here the prediction model for the non-specific portion of the sorption isotherm of anionic PFAS was used (eq. 7) (Qin *et al.*, 2024). The *K*_BSA/w_ was fixed at 1.31 for very hydrophilic chemicals at log *K*_ow_ < 2 (deBruyn *et al.*, 2007).


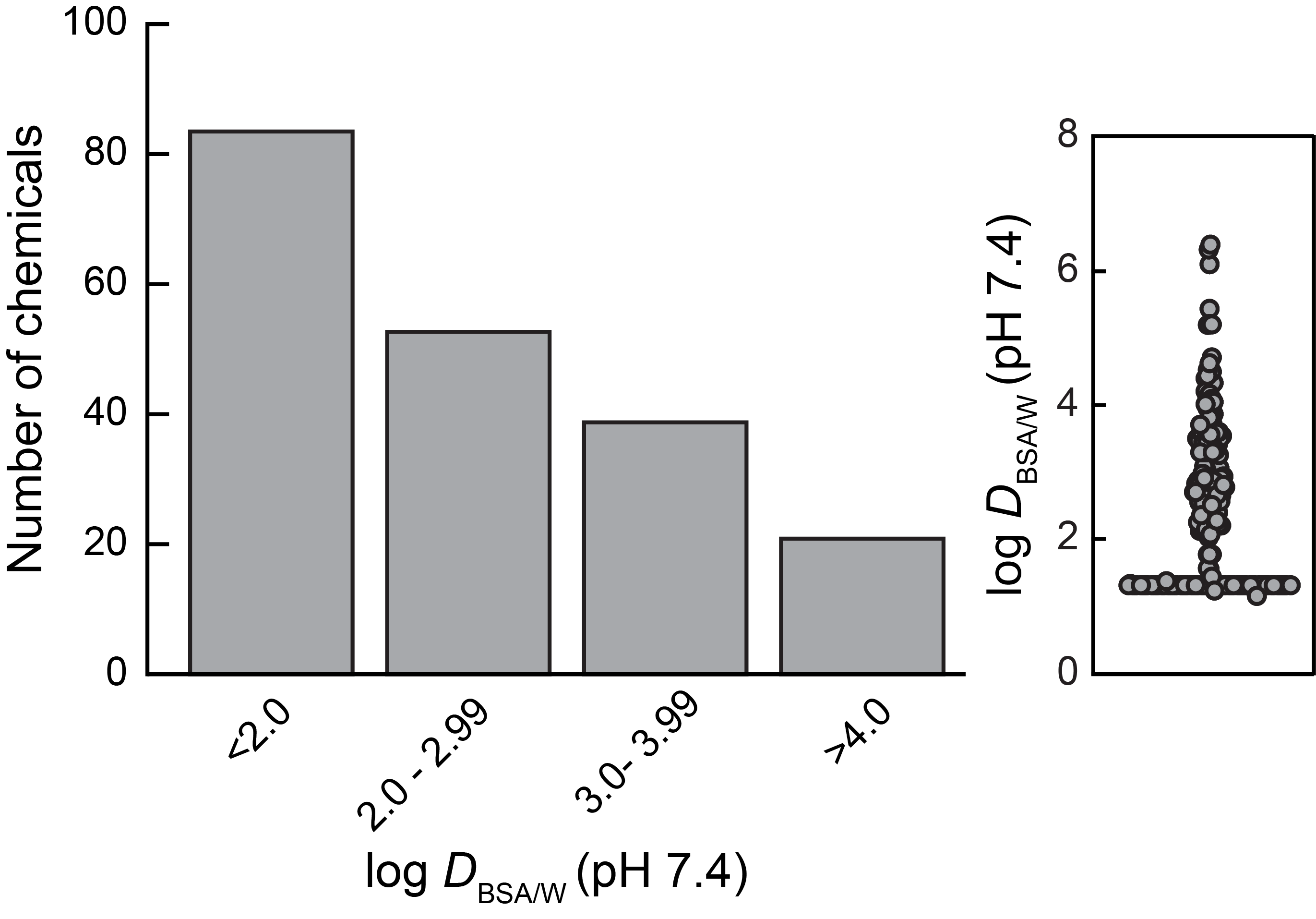


$\text{log }\text{K}_{\text{BSA/w}}\text{(neutral)=0.70×log }\text{D}_{\text{lip/w}}\text{+0.34}$ (6)

$\text{log }\text{D}_{\text{BSA/w}}\text{(anion}\text{ic}\text{)=0.75×log }\text{D}_{\text{lip/w}}\text{+1.01}$ (7)

The proteins in cells are rather like structural proteins (SP) and if no experimental data or LSER parameters were available, the *K*_SP/w_ was predicted from *K*_ow_ with eq. 8 (Endo *et al.*, 2012). The binding of anions to SP is weaker than to BSA (Henneberger *et al.*, 2016), and we used the analogous equation to eq. 3 to predict *D*_SP/w_(pH) (eq.8).

$\text{log }\text{K}_{\text{SP/w}}\text{(neutral)=0.73×log }\text{D}_{\text{lip/w}}\text{-0.93}$ (8)

$\text{D}_{\text{SP/w}}\left( \text{pH} \right)\text{=}\text{f}_{\text{neutral}}\text{×}\text{K}_{\text{SP/w}}\text{(neutral)}\text{ + (1- }\text{f}_{\text{neutral}}\text{)}\text{×}\frac{\text{K}_{\text{SP/w}}\text{(neutral)}}{10}$ (9)

References

ACD/Percepta (2015). Build 2726, Advanced Chemistry Development, Inc., Toronto, On, Canada, [*http://www.acdlabs.com*](http://www.acdlabs.com).

Adamovsky, O., Groh, K. J., Białk-Bielińska, A., Escher, B. I., Beaudouin, R., Mora Lagares, L., Tollefsen, K. E., Fenske, M., Mulkiewicz, E., Creusot, N., et al. (2024). Exploring BPA alternatives – Environmental levels and toxicity review. Environ Int **189**, 108728.

deBruyn, A. M. H., and Gobas, F. A. P. C. (2007). The sorptive capacity of animal protein. Environ Sci Technol **26**(9), 1803-1808.

Endo, S., Bauerfeind, J., and Goss, K.-U. (2012). Partitioning of Neutral Organic Compounds to Structural Proteins. Environ Sci Technol **46**(22), 12697-12703.

Endo, S., Escher, B. I., and Goss, K. U. (2011a). Capacities of Membrane Lipids to Accumulate Neutral Organic Chemicals. Environ Sci Technol **45**(14), 5912-5921.

Endo, S., and Goss, K. U. (2011b). Serum Albumin Binding of Structurally Diverse Neutral Organic Compounds: Data and Models. Chem Res Toxicol **45**(24), 2293-2301.

Escher, B., Abagyan, R., M, E., Klüver, N., Redman, A., Zarfl, C., and Parkerton, T. (2020). Recommendations for improving methods and models for aquatic hazard assessment of ionizable organic chemicals. Environ Sci Technol **39**(1), 269-286.

Escher, B. I., and Sigg, L. (2004). Chemical Speciation of Organics and of Metals at Biological Interfaces. In Physicochemical Kinetics and Transport at Biointerfaces (H. P. Van Leeuwen, and W. Köster, Eds.), Vol. 9, pp. 205 - 271. John Wiley & Sons, Chichester.

Henneberger, L., Goss, K. U., and Endo, S. (2016). Partitioning of Organic Ions to Muscle Protein: Experimental Data, Modeling, and Implications for in Vivo Distribution of Organic Ions. Environ Sci Technol **50**(13), 7029-7036.

Huchthausen, J., Braasch, J., Escher, B. I., König, M., and Henneberger, L. (2024). Effects of Chemicals in Reporter Gene Bioassays with Different Metabolic Activities Compared to Baseline Toxicity. Chem Res Toxicol **37**(5), 744-756.

Niu, L., Henneberger, L., Huchthausen, J., Krauss, M., Ogefere, A., and Escher, B. I. (2022). pH-Dependent Partitioning of Ionizable Organic Chemicals between the Silicone Polymer Polydimethylsiloxane (PDMS) and Water. ACS Environmental Au doi: 10.1021/acsenvironau.1c00056.

Qin, W., Henneberger, L., Glüge, J., König, M., and Escher, B. I. (2024). Baseline Toxicity Model to Identify the Specific and Nonspecific Effects of Per- and Polyfluoroalkyl Substances in Cell-Based Bioassays. Environ Sci Technol **58**(13), 5727-5738.

Ulrich, N., Endo, S., Brown, T. N., Watanabe, N., Bronner, G., Abraham, M. H., and Goss, K.-U. (2021). UFZ-LSER database v 3.2.1 [Internet], Leipzig, Germany, Helmholtz Centre for Environmental Research-UFZ. 2017 [accessed on 21 March 2021]. Available from <http://www.ufz.de/lserd>.

US EPA (2023). CompTox Chemicals Dashboard, <https://comptox.epa.gov/dashboard/> accessed on 20 Dec 2023.

Williams, A. J., Lambert, J. C., Thayer, K., and Dorne, J. C. M. (2021). Sourcing data on chemical properties and hazard data from the US-EPA CompTox Chemicals Dashboard: A practical guide for human risk assessment. Environ Int **154**, 106566.
